# Evaluation of pathogenicity variation between two *Erwinia* species in apples and their population using a duplex real-time PCR method

**DOI:** 10.3389/fmicb.2025.1514551

**Published:** 2025-02-28

**Authors:** Mi-Hyun Lee, Kotnala Balaraju, Hyo-Won Choi, Yong Hwan Lee

**Affiliations:** ^1^Crop Protection Division, National Institute of Agricultural Sciences, Wanju, Republic of Korea; ^2^Disaster Management Division, Rural Development Administration, Jeonju, Republic of Korea

**Keywords:** fire blight, black shoot blight, apple trees, duplex real-time PCR, temperature variation, swarming motility

## Abstract

Fire blight and black shoot blight diseases, caused by *Erwinia amylovora* and *Erwinia pyrifoliae*, respectively, continue to spread several areas in Korea, despite intensive efforts by the government to control diseases. The distribution pattern of fire blight and black shoot blight is different from each other in Korea. Consequently, it is required to investigate the pathogenicity of *E. amylovora* and *E. pyrifoliae* in apple trees. The disease severity of fire blight and black shoot blight was compared in this study by an artificial inoculation of *E. amylovora* and *E. pyrifoliae* suspensions into the abaxial veins of apple leaves and measuring their pathogenicity at varying temperatures. Furthermore, disease severity was assessed by inoculating *E. amylovora* and *E. pyrifoliae* in apple flowers and assessing their pathogenicity at various temperatures. The *E. amylovora*-inoculated flowers displayed greater disease index than *E. pyrifoliae*-inoculated flowers at temperatures ranging from 18°C to 25°C. Upon examining the population sizes of *E amylovora* and *E. pyrifoliae* in flowers using a real-time polymerase chain reaction (PCR), the Ct value of *E. amylovora* was found to be lower in the style including stigma and hypanthium than the Ct value of *E. pyrifoliae*, except at 18°C. Hypanthium contained *E. amylovora* TS3128 and *E. pyrifoliae* YKB12327 at >10^7^ and 10^5^ CFU/mL, respectively at 15°C. Furthermore, in this study, we investigated the population size of *E. amylovora* and *E. pyrifoliae* in apple flowers in relation to temperature in order to clarify the differences in their pathogenicity.

## Introduction

Fire blight is one of the most destructive bacterial diseases of several fruit crops, including apples, pears and other rosaceous plants; it is caused by the pathogen *Erwinia amylovora* (Medhioub et al., 2022). Due to the severity of its pathogenicity, it has become the global concern, resulting in reduced agricultural output. Fire blight was identified in apples, Asian pears, and Chinese quince in Korea in 2015 (Park et al., 2016; Myung et al., 2016). In 2020, fire blight occurred in 744 orchards in Korea. Fire blight symptoms appear in all plant organs, such as blossoms, shoots, twigs, fruits, and the rootstock near the graft union on the lower trunk (Eastgate, 2000). The infected tissue is observed to turn brown to black, resulting in gradual death (Jung et al., 2023). It has been reported that infected flowers also become a pathway for the expansion of the disease (Kharadi et al., 2021). Most recently, blight symptoms were observed in Chinese hawthorn (*Crataegus pinnatifida*) caused by *E. amylovora* in Korea (Lim et al., 2023), and an apricot (*Prunus armeniaca*) orchard during an outbreak of fire blight that had affected a nearby apple orchard (Lee et al., 2021). The pathogen can infect all host tissues at various times during the season, making it challenging to control fire blight (Santander and Biosca, 2017).

*Erwinia pyrifoliae*, another species within the genus *Erwinia*, is a necrotrophic pathogen that causes black shoot blight in Asian pears (*Pyrus pyrifolia*) in Korea. Initially, the pathogen was consistently isolated from orchards in Chuncheon in 1995. It was then inoculated on agar plates and then identified through morphological and molecular means (Kim et al., 1999; Rhim et al., 1999). Black shoot blight was first observed in pear trees in Korea; however, it has also been shown to affect apple trees (Han et al., 2016). *E. pyrifoliae* has only been detected in Korea, with the exception of strawberries in the Netherlands (Wenneker and Bergsma-Vlami, 2015). The symptoms of black shoot blight in pear trees appear blackish blight in twigs and black stripes on leaves (Kim et al., 1999; Lee et al., 2020). Over the last decade, black shoot blight caused by *E. pyrifoliae* has mostly occurred in apple trees (Lee et al., 2023).

The morphological, cultural, and biochemical characteristics of *E. pyrifoliae* were similar to those of *E. amylovora* (Kim et al., 1999; Shrestha et al., 2003); however, there are temperature-driven differences in the growth rates of the two species. Specifically, *E. pyrifoliae* is more cold-tolerant than *E. amylovora* (Shrestha et al., 2005). Additionally, some studies have presented molecular evidence regarding the differences between *E. pyrifoliae* and *E. amylovora* (Jock and Geider, 2004; McGhee et al., 2002).

*Erwinia amylovora* can infect various parts of trees, including flowers, shoots, and rootstocks. Its infection of the flowers, known as blossom blight, is the most important within the context of disease outbreak and management (Bubán et al., 2003). This is because the flowers provide a nutrient-rich and moist environment suitable for the epiphytic growth of *E. amylovora* (Norelli and Brandl, 2004). In flower infection, *E. amylovora* occurs the rapid multiplication in the stigma and then moves down toward the hypanthium. Once the flowers have been infected, the pathogens quickly spread to the branches and other parts of the tree (Cui et al., 2021). In a previous study, it has been reported that the population size of *E. pyrifoliae* on the surface of pear blossoms was significantly smaller than the population size of *E. amylovora*, which suggests that *E. pyrifoliae* does not grow as optimally as does *E. amylovora* on pear blossoms at 23°C, as determined using real-time PCR (Lehman et al., 2008).

Since the detection of fire blight in Korea in 2015, *E. amylovora* and *E. pyrifoliae* have been detected simultaneously different outbreaks. Fire blight and black shoot blight have spread to several areas of Korea, despite intensive efforts by the Korean government to restrict their spread (Choi et al., 2022). The pattern of distribution and occurrence of fire blight and black shoot blight was different in Korea. Therefore, studying the pathogenicity of *E. amylovora* and *E. pyrifoliae* in apple trees is crucial. In this study, differences in the dynamics of the two diseases are found to be due to differences in the pathogenicity of the two pathogens in apple trees. In particular, the movement and proliferation of the two pathogens in apple flowers were affected by temperature, and the population sizes in apple flowers was lower than of *E. pyrifoliae* in not only style including stigma but also hypanthium.

## Materials and methods

### Distribution of fire blight and black shoot blight in apples in Korea

A survey was conducted to investigate the occurrence of fire blight from 2015 to 2022 and black shoot blight disease from 1995 to 2022 in apple orchards in Korea. The following are the 28 localities from 6 provinces in which the survey for the occurrence of fire blight disease was conducted from 2016 to 2021: Eleven places in Gyeonggi (Yeoncheon, Paju, Yangju, Namyangju, Gwangju, Yongin, Icheon, Pyeongtaek, Anseong, Yeoju, and Hwaseong), four places in Gangwon (Pyeongchang, Wonju, Yeongwol, and Hongcheon), six places in Chungbuk (Jecheon, Chungju, Eumseong, Danyang, Jincheon, and Goesan), four places in Chungnam (Cheonan, Dangjin, Yesan, and Asan), two places in Gyeongbuk (Yeongju and Andong), and one place in Jeonbuk (Iksan). The survey for the occurrence of black shoot blight was conducted from 1995 to 2022 in 5 provinces, 26 cities which are as follows: nine places in Gyeonggi (Gapyeong, Pocheon, Yangpyeong, Namyangju, Yangju, Goyang, Anseong, Gwangju, and Yeoncheon), eleven places in Gangwon (Wonju, Hongcheon, Yanggu, Hoengseong, Yeongwol, Cheorwon, Chuncheon, Hwacheon, Pyeongchang, Goseong, and Inje), three places in Chungbuk (Jecheon, Chungju, and Eumseong), one place in Chungnam (Cheonan), and two places in Gyeongbuk (Yeongju and Mungyeong). For the disease occurrence analysis, the suspected samples were collected from orchards of various places and tested for the disease outbreak.

### Measurement of motility at different temperatures

The bacterial pathogens, such as *E. amylovora* TS3128 and *E. pyrifoliae* YKB12327 used in this study were isolated from infected pear and apple trees in Korea in 2015 (Park et al., 2018); these samples were plated on King’s B and Luria Bertani (LB) media, respectively, and cultured at 27°C for 48 h. Bacterial cell suspensions were prepared by diluting in sterile distilled water (SDW) until the optical density at 600 nm (OD_600_) reached 1.0. Motility was assessed following the method described by Edmunds et al. (2013), with some modifications. Briefly, 10 μL of overnight grown *E. amylovora* TS3128 and *E. pyrifoliae* YKB12327 cultures were stab-inoculated onto 0.3% LB agar plates, and incubated at various temperatures, including 14°C, 18°C, and 27°C for 48 h. The diameter traveled from the inoculation site was measured using analog calipers (Hitec, Germany). The assay was repeated at least once in triplicates.

### Comparison of the pathogenicity of two pathogens in apple fruits and leaves

The pathogenicity test was carried out on apple fruits following the method described by Lee et al. (2021). Before inoculation, bacterial suspensions of *E. amylovora* TS3128 and *E. pyrifoliae* YKB12327 cultured at 27°C for 48 h in LB medium were suspended with SDW and adjusted to a concentration of 1.5 × 10^8^ CFU/mL. For the pathogenicity test on fruits, healthy immature apple (*Malus domestica* cv. Fuji) fruits were surface-disinfected with 70% ethanol for 30 s, and rinsed with SDW. The fruits were then dried and wounded by piercing them 2 to 3 mm deep with a sterile pin. The incision site was then inoculated with 10 μL bacterial suspensions (1.5 × 10^8^ CFU/mL). The inoculated fruits were placed in a plastic box containing moist paper to maintain the humidity, and incubated for 10 days at various temperatures, such as 12°C, 18°C, 21°C, and 27°C. The symptoms, such as necrosis and bacterial ooze out observed at inoculated sites by two bacteria, *E. amylovora* TS3128 and *E. pyrifoliae* YKB12327 were compared with non-inoculated fruits. The pathogenicity tests were repeated at least once in triplicates.

For the pathogenicity test on leaves, the upper third and fourth leaves on apple shoot were collected from the greenhouse at the National Institute of Agricultural Sciences (NAS). The petioles of the abaxial side of apple leaves were punctured with a sterile needle and inoculated with 2 μL of each bacterial suspension. The inoculated leaves were placed in a square plate containing moist tissue paper and incubated the plates at 14°C, 16°C, and 18°C for 15 days, and then leaves were monitored for the development of disease symptoms. Disease symptoms were scored using a disease index scale ranging from 0 to 5 ratings (where 0 = no symptoms, 1 = lesions with <10% disease incidence, 2 = lesions with 11 to 20%, 3 = lesions with 21 to 40%, 4 = lesions with 41 to 70%, 5 = symptoms with >71 to 100%). The disease ratings on apples leaves were explained in Supplementary Figure S1A. The experiment was repeated at least once in triplicates.

### Comparison of the pathogenicity of two pathogens in apple flowers

One year old crab potted apple (*Malus sieboldii*) plants were purchased from the local market and grown under greenhouse conditions at NAS until flowerings. Flowers of similar size were detached from the potted plants and each flower was placed in a single Eppendorf vial (2 mL) containing SDW. After 24 h, all the flowers were inoculated with bacterial suspensions (10^6^ CFU/mL) of *E. amylovora* TS3128 and *E. pyrifoliae* YKB12327; approximately 0.2 μL of inoculum was applied per flower by touching a droplet to each stigma (i.e., normally 3–4 per flower). Fifteen flowers from each treatment were maintained in a plastic box and assessed after 5 days. Disease symptoms were scored using a disease index scale ranging from 0 to 5 ratings (where 0: no necrosis; 1: necrosis on the stigma; 2: necrosis visible on the stigma and hypanthium; 3: necrosis extending into the ovary, no farther than the widest point; 4: necrosis extending to the base of the ovary; 5: necrosis extending into the peduncle). The disease ratings on apple flowerbuds were explained in Supplementary Figure S1B.

### Duplex real-time PCR for quantification of *E. amylovora* and *E. pyrifoliae* populations

After assessing the disease severity, stigmas of all flowers were collected along with portions of the supporting styles. The hypanthium was partially isolated by removing the petals, calyx, and pedicel. The stigma and hypanthium of the sampled style were placed in a sterile 1.5 mL micro centrifuge tubes containing 100 and 1,000 μL sterile buffer (10 mM potassium phosphate, pH 7.0), respectively. The samples were macerated and incubated at 23°C for 30 min. The experiment was performed twice with five replicates (flowers). The template for real-time PCR was directly derived from the processed sample lysis. Duplex real-time PCR using a HelixDtec™ EAEP detection kit (Cat no. EAEP-T100, Nanohelix, Daejeon, Korea) was used to compare *E. amylovora* and *E. pyrifoliae* populations in style, stigma and hypanthium. Real-time PCR was performed according to the manufacturer’s instructions using the Bio-Rad CFX maestro software (BioRad, Hercules, CA, United States). This experiment was performed at least three times.

### Statistical analysis

Statistical analyses were performed using the R software (ver. 4.2.3.). Data were analyzed using either Student’s *t*-test (normality test passed) or the Mann–Whitney Rank sum test (normality test failed). A *p*-value of <0.05 was considered statistically significant.

## Results

### Distribution of fire blight and black shoot blight in Korea

**Figure 1:**
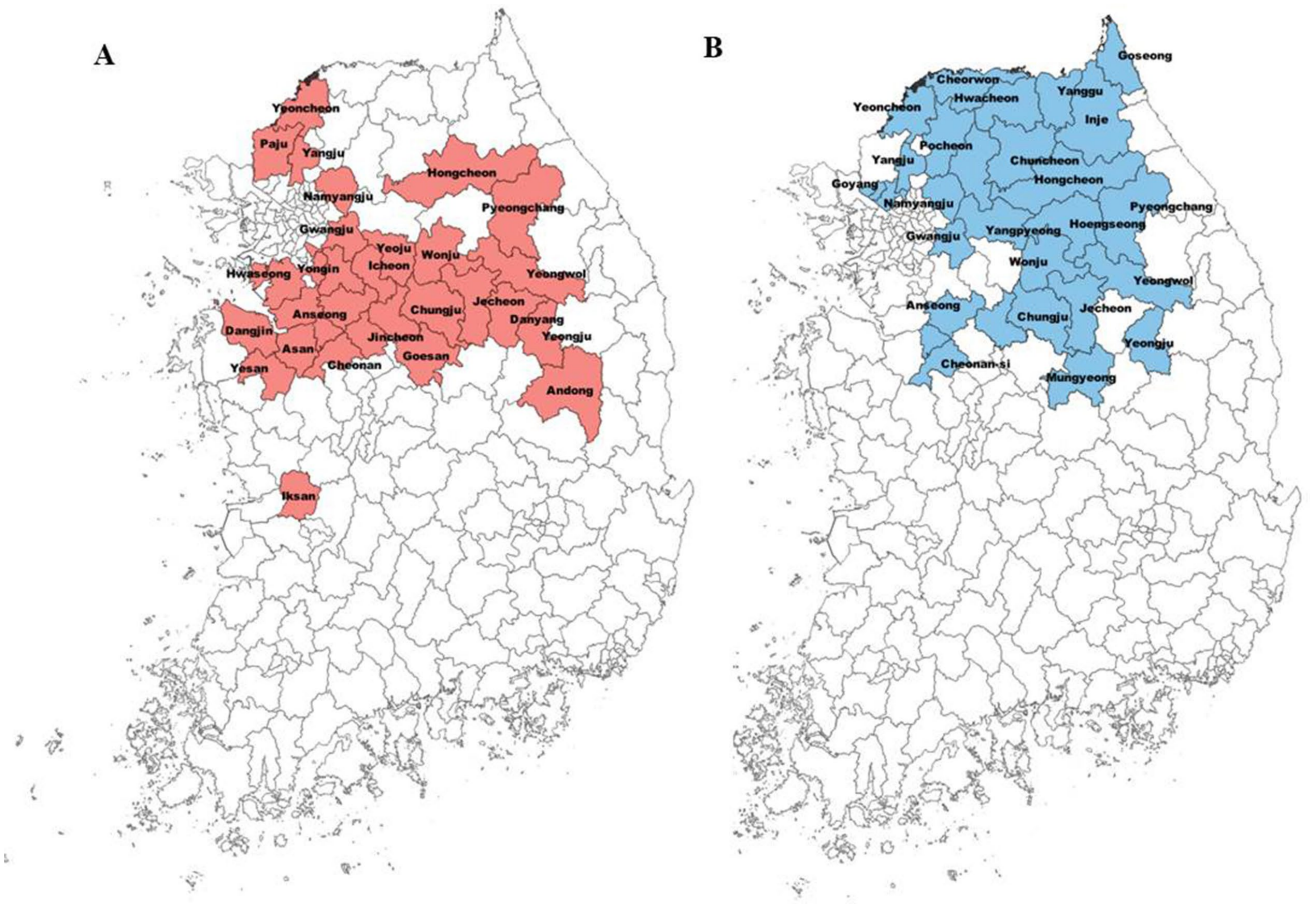
Distribution of fire blight and black shoot blight among cities in Korea. The colored ones have high occurrence of these diseases. Red and blue colors indicate the cities where fire blight **(A)** and black shoot blight **(B)** were reported, respectively.

Since the 2015 outbreak of the fire blight in Korea, it has been spread to 28 cities in 6 provinces (Figure 1A). The distribution pattern of the fire blight indicates that it has been primarily disseminated in central regions. On the other hand, black shoot blight, an endemic pathogen, has been spread in 26 cities in five provinces over 20 years (Figure 1B). Black shoot blight was distributed throughout the northern region, with the exception of Yeongju and Mungyeong (Figure 1B).

### Effect of different temperatures on motility of pathogens

**Figure 2:**
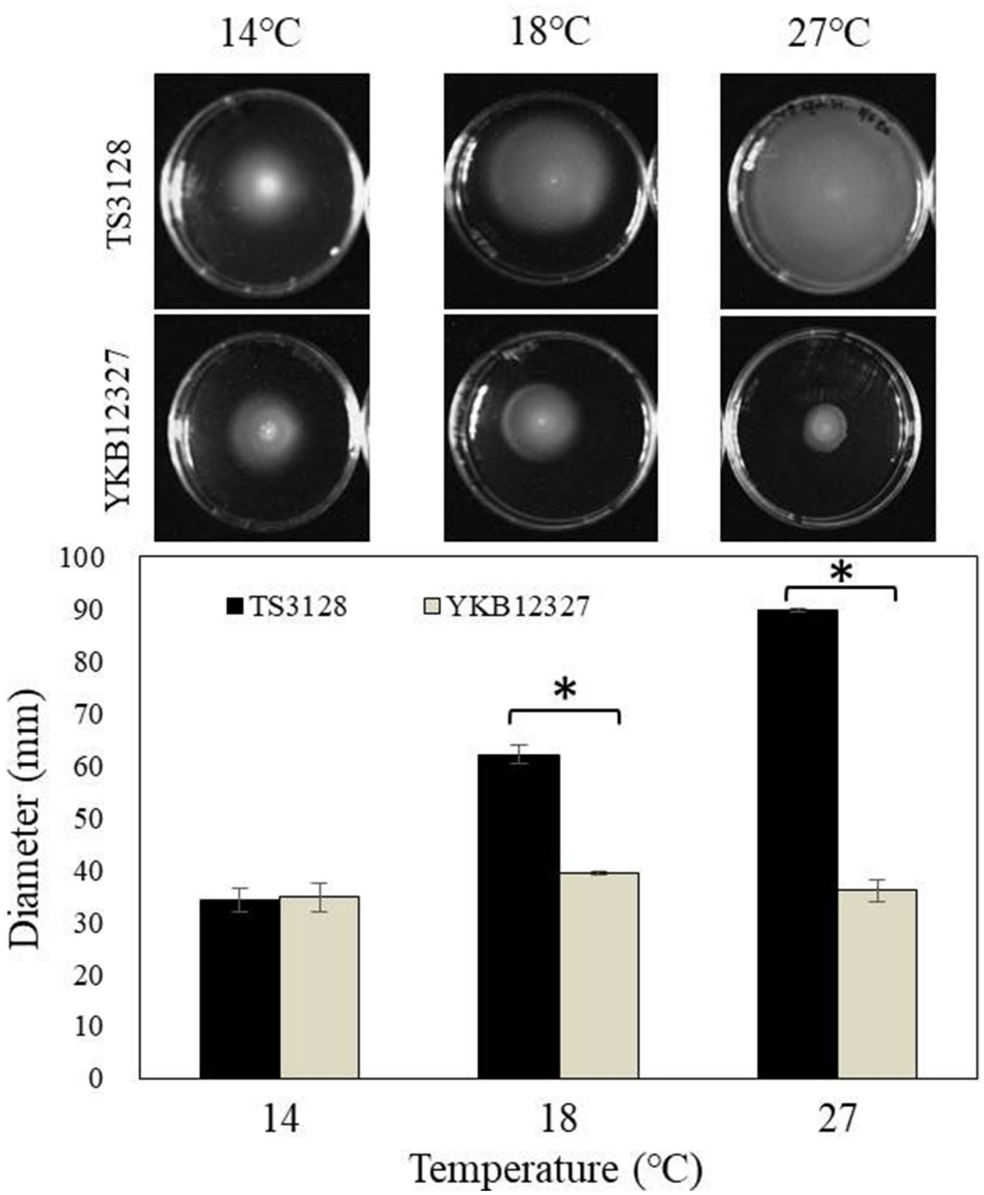
Effect of different temperatures on motility of two pathogens (*E. amylovora* TS3128 and *E. pyrifoliae* YKB12327). The plates contained semi-solid LB medium with 0.3% agar were inoculated with bacterial suspensions of *E. amylovora* TS3128 and *E. pyrifoliae* YKB12327. Diameters of the motility of *E. amylovora* TS3128 (black bar) and *E. pyrifoliae* YKB12327 (gray bar) in semi-solid medium were recorded 48 h after incubation at 14°C, 18°C, and 27°C. The experiment was repeated once with triplicates. Asterisks indicate significant differences (*p* < 0.05) based on Student’s *t*-test.

When two pathogens were cultured at different temperatures, such as 14°C, 18°C, and 27°C, *E. amylovora* TS3128 has been displayed to exhibit a greater motility than the pathogen *E. pyrifoliae* at all temperatures (Figure 2). However, the highest motility in *E. amylovora* has been observed as 89.67 mm in diameter at 27°C, while the motility of other pathogen *E. pyrifoliae* YKB12327 was only 36 mm in diameter 48 h after incubation. The motility of *E. amylovora* TS3128 was increased with increasing temperature; while the motility of *E. pyrifoliae* YKB12327 reduced with increasing temperature (Figure 2). Whereas no growth was observed in the control plates.

### Comparison of the pathogenicity of two pathogens at different temperatures

**Figure 3:**
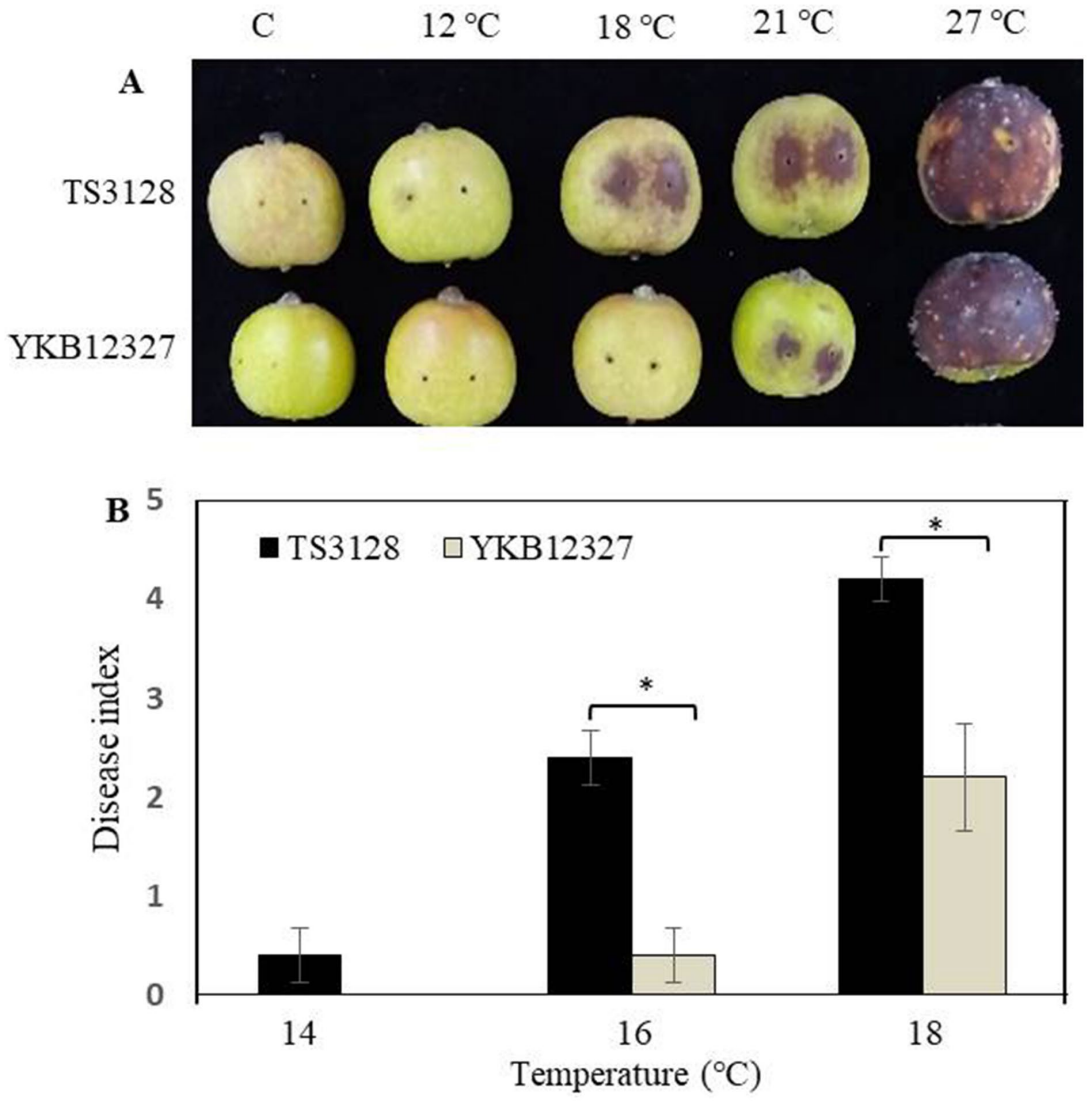
Effect of temperature on pathogenicity of *E. amylovora* TS3128 and *E. pyrifoliae* YKB12327 on apple fruits and leaves by an artificial inoculation. **(A)** The development of disease symptoms on wounded apple fruits 10 d after inoculation (dai) was characterized by necrosis and browning followed by rotting, while no disease symptoms were developed on non-inoculated fruits (control). **(B)** Disease index in apple leaves infected with *E. amylovora* TS3128 and *E. pyrifoliae* at different temperatures (14°C, 16°C, and 18°C). The disease symptoms were developed on inoculated leaves, and no symptoms were developed on non-inoculated leaves. The experiment was repeated at least once in triplicates. Asterisks indicate significant differences (*p* < 0.05) based on Student’s *t*-test.

The pathogenicity of two pathogens *E. amylovora* TS3128 and *E. pyrifoliae* YKB12327 was tested on two plant materials, such as immature apple fruits and leaves at different temperatures ranging from 12°C to 27°C. *E. amylovora* TS3128 exhibited more severe symptoms at temperatures ranging from 18°C to 21°C in immature apple fruits compared to *E. pyrifoliae* YKB12327, where the symptoms started to appear at 21°C (Figure 3A). The pathogenicity of *E. amylovora* TS3128 differs from that of *E. pyrifoliae* YKB12327 at 18°C. The pathogenicity assay demonstrated that both bacteria caused severe symptoms, with the symptoms being most severe at 27°C. To compare the pathogenicity between *E. amylovora* TS3128 and *E. pyrifoliae* YKB12327 on apple leaves at low temperatures, the disease index scale ranging from 0 to 5 was developed based on disease severity score (0–100%) (Figure 3B). Disease index on apple leaves by the pathogen *E. amylovora* TS3128 has been found to show higher than the disease index by *E. pyrifoliae* YKB12327 at temperatures 16 and 18°C (Figure 3B). The symptoms corresponding to disease severity of *E. amylovora* TS3128 were indicated by an index of more than 4.2 at 18°C. In contrast, the disease index for *E. pyrifoliae* YKB12327 has been shown to be 2.2. On the other hand, no symptoms were developed on non-inoculated (control) apple fruits.

### Effect of temperature on pathogenicity of two pathogens on apple flowers

**Figure 4:**
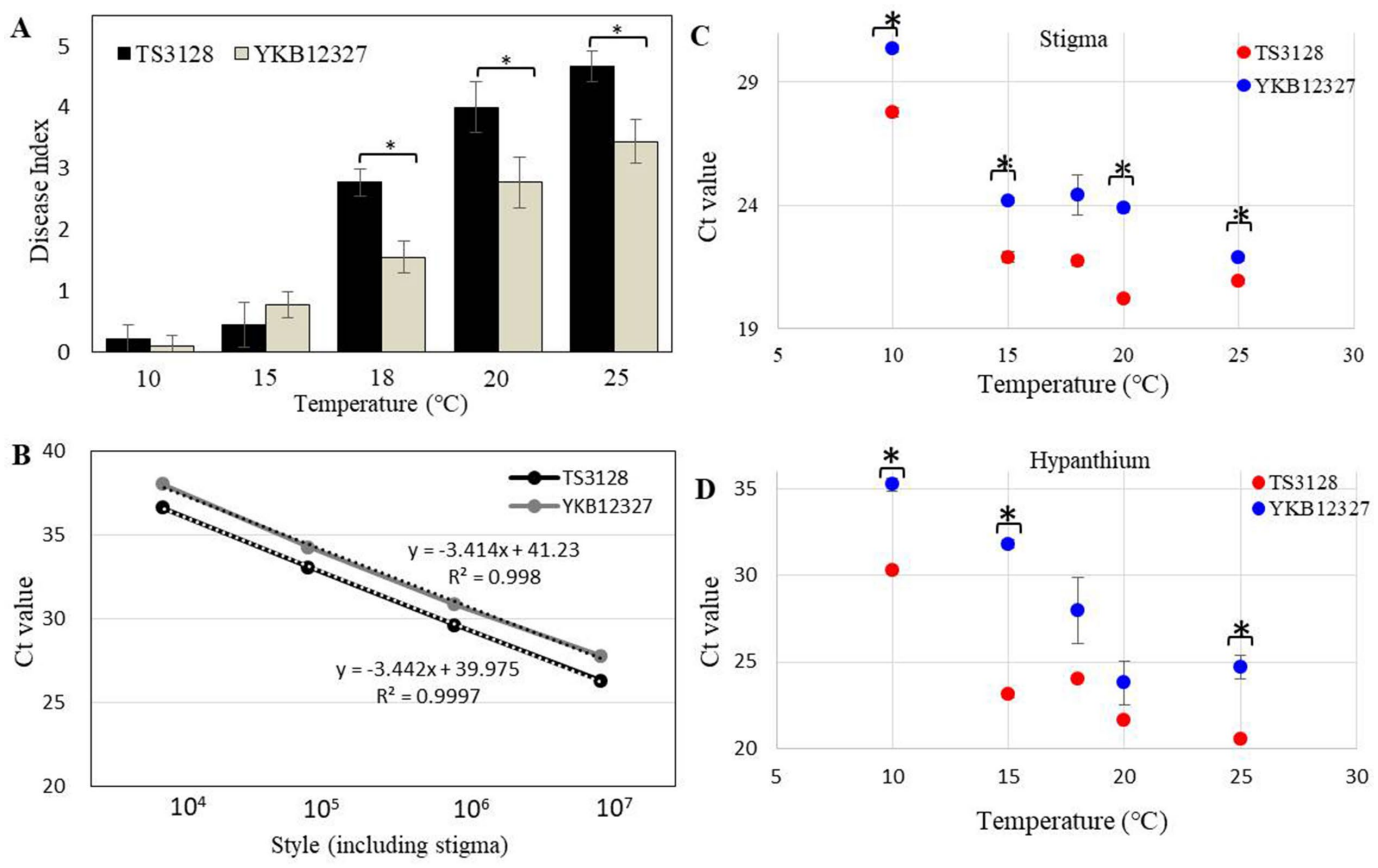
Disease index in apple flowers inoculated with *E. amylovora* TS3128 and *E. pyrifoliae* YKB12327 and their population size using a duplex real-time PCR. **(A)** Disease index in apple flowers inoculated with *E. amylovora* TS3128 and *E. pyrifoliae* YKB12327 at different temperatures ranging from 10°C to 25°C. The experiment was repeated at least once in triplicates. **(B)** Standard curve for real-time PCR of *E. amylovora* TS3128 and *E. pyrifoliae* YKB12327 (serially diluted (10^7^ to 10^4^) in water). The *R*^2^ value of each curve was >0.99. Population size of *E. amylovora* TS3128 and *E. pyrifoliae* YKB12327 on the styles of apple flowers, including the stigma **(C)** and hypanthium **(D)**, assessed using a real-time PCR. Red and blue shading indicates fire blight and black shoot blight, respectively. Asterisks indicate significant differences (*p* < 0.05) based on Student’s *t*-test.

When the stigma of the flower was infected with *E. amylovora* TS3128 and *E. pyrifoliae* YKB12327 pathogen suspensions, the infection progressed through the style. Disease symptoms were developed more on stigmas inoculated with *E. pyrifoliae* YKB12327 than in *E. amylovora* TS3128-inoculated stigmas, which implies that *E. pyrifoliae* YKB12327 also causes equal damage to the crop as that of *E. amylovora* TS3128. However, when the pathogenicity of *E. amylovora* TS3128 and *E. pyrifoliae* YKB12327 on apple flowers were compared at different temperatures, the virulence of the *E. amylovora* TS3128 was found to be greater than the virulence of the pathogen *E. pyrifoliae* YKB12327 on stigmas of the apple flowers at all temperatures (10 to 25°C). The flowers inoculated with *E. amylovora* TS3128 and *E. pyrifoliae* YKB12327 showed increased disease symptoms at 18°C and higher temperatures (Figure 4A). The flowers inoculated with *E. amylovora* TS3128 showed a higher disease index than those infected with *E. pyrifoliae* YKB12327, while no symptoms were observed in the non-inoculated (control group) apple flowers.

### Establishment of the duplex real-time PCR for quantification and population of two pathogens on flowers at different temperatures

The duplex standard curves for *E. amylovora* TS3128 and *E. pyrifoliae* YKB12327 are shown in Figure 4B. The mean threshold cycle (Ct) value (*n* = 3) was plotted against the density of bacterial cells (10^4^–10^7^ CFU/mL) to construct standard curves (Table 1). The Ct values of *E. amylovora* TS3128 and *E. pyrifoliae* YKB12327 cells (10^7^ CFU/mL) were 26.27 and 27.74, respectively. The Ct value in the real-time PCR increases as the population density of bacterial cells decreases.

**Table 1:** Mean threshold cycles (Ct) of 10-fold dilution series of *E. amylovora* TS3128 and *E. pyrifoliae* YKB12327 cell suspensions using a real-time PCR.

*E. amylovora* TS3128 cell suspensions		*E. pyrifoliae* YKB12327 cell suspensions	
CFU/mL reaction mix	Ct ± SD^a^ (*n* = 3)	CFU/mL reaction mix	Ct ± SD^a^ (*n* = 3)
7.0 × 10^7^	26.27 ± 0.02	3.5 × 10^7^	27.74 ± 0.00
7.0 × 10^6^	29.59 ± 0.09	3.5 × 10^6^	30.83 ± 0.07
7.0 × 10^5^	33.02 ± 0.01	3.5 × 10^5^	34.22 ± 0.02
7.0 × 10^4^	36.60 ± 0.32	3.5 × 10^4^	37.99 ± 0.26
7.0 × 10^3^	N.D.^b^	3.5 × 10^3^	N.D.^b^

a SD, standard deviation of three reactions.

b N.D., not determined.

The Ct value of *E. amylovora* TS3128 was found to be lower than that of *E. pyrifoliae* YKB12327 in the style, which included stigma and hypanthium, except at 18°C, when the population sizes of *E. amylovora* TS3128 and *E. pyrifoliae* YKB12327 in apple flowers were compared using the real-time PCR (Figure 4C). The style including stigma was cultured at 20°C after inoculation, and the maximal difference in population size between *E. amylovora* TS3128 and *E. pyrifoliae* YKB12327 was observed. However, the two pathogens did not exhibit statistically significant difference in their population sizes in the hypanthium (Figure 4D). Nevertheless, *E. pyrifolia* YKB12327 exhibited a substantial lower population size in hypanthium at 15°C and lower temperatures than *E. amylovora* TS3128. Moreover, the population size of *E. amylovora* TS3128 in the hypanthium was >10^7^ CFU/mL, while that of *E. pyrifoliae* YKB12327 was approximately 10^5^ CFU/mL at 15°C. Consequently, the two pathogens exhibited a population size divergence of approximately 10^2^ CFU/mL at 15°C.

## Discussion

In this study, we discuss the variations in pathogenicity between *E. amylovora* and *E. pyrifoliae*, which cause fire blight and black shoot blight, respectively. These two diseases have resulted in severe economic losses in Korea by destroying a substantial quantity of fruits that could have been exported (Park et al., 2017). The number of orchards infected with fire blight is greater than that of orchards infected with black shoot blight, despite the fact that fire blight first appeared in Korea 20 years prior to black shoot blight (Ham et al., 2020; Lee and Lee, 2022). Furthermore, fire blight disease spreads more rapidly in the central region rather than in the northern region; nevertheless, black shoot blight is more prevalent in the northern region, which has a relatively low average temperature. Although the phenotypical characteristics of *E. pyrifoliae* are similar to those of *E. amylovora*, a detailed study has demonstrated that these two pathogens differ from each other; with *E. pyrifoliae* having greater cold tolerance than *E. amylovora* (Shrestha et al., 2005).

In 2020, 744 orchards in Korea were affected by fire blight, 73% of 744 orchards were apple orchards, while the remaining 27% were pear orchards (Lee et al., 2022). Over the last decade, black shoot blight caused by *E. pyrifoliae* has mostly affected apple trees (Choi et al., 2022). Apple trees infected with fire blight and black shoot blight may support the rapid growth of the pathogen. Therefore, it is necessary to study the ecological aspects such as the pathogenicity of *E. amylovora* and *E. pyrifoliae* on apple trees. *E. amylovora* TS3128 has been found to grow faster than *E. pyrifoliae*. Our findings imply that swimming motility on a semi-solid medium contributes to the virulence of *E. amylovora* TS3128 at 27°C. Our finding are corroborated by a recent study (Tao et al., 2023), which stated that motility was identified as a pathogenic characteristics of *Erwinia sorbitol* sp. Nov. Similarly, a previous study by Hossain et al. (2005) demonstrated that the motility of *Erwinia carotovora* played a role in its virulence on tobacco leaves. Conversely, Kharadi et al. (2019) have reported that pathogens with low amylovoran production were not phytopathogenic. The motility of bacteria might be attributed to the formation of flagella, which played a vital role in the survival of the bacteria upon production of amylovoran for the attachment to host cell surfaces (Santander et al., 2014).

Temperature is known as one of the environmental parameters required to achieve the high cell density needed for flower infection (MAAARO, 2011). According to Santander and Biosca (2017), the ideal temperature for the growth of *E. amylovora* is 28°C. However, it was recently shown that the pathogenicity of *E. amylovora* persists even at lower temperatures (14°C and 4°C). Previously, Shrestha et al. (2005) reported that *E. pyrifoliae* grew faster at lower temperatures ranging from 12 to 21°C than *E. amylovora*. The difference in growth rate at these temperatures indicated that *E. pyrifoliae* is more cold-tolerant than *E. amylovora*. In general, rising temperatures encourage the growth and spread of plant diseases, particularly fungi (Pour et al., 2020) and bacteria (Devi et al., 2022), while also affecting host defensive mechanisms. In our study, the growth of *E. amylovora* was higher than that of *E. pyrifoliae*; and it exhibited higher level of pathogenicity in immature apple fruits.

In order to compare the pathogenicity of *E. amylovora* and *E. pyrifoliae* on flowers, it is necessary to investigate the population size of the two pathogens. In recent years, the real-time PCR method has been employed as a simple and sensitive method for the detection of *E. amylovora* and *E. pyrifoliae* (Ham et al., 2022). In particular, the real-time PCR diagnostic and droplet digital polymerase chain reaction (ddPCR) kits for detecting *E. amylovora* and *E. pyrifoliae*, have been commercialized in Korea (He et al., 2023). The population size of *E. pyrifoliae* was significantly smaller than that of *E. amylovora* in apple flowers at temperatures between 10 and 25°C. This is in consistent with a recent study by Choi et al. (2022), in which *E. amylovora* was shown to have larger colony numbers than *E. pyrifoliae* when co-cultured in liquid media and co-inoculated into immature apple fruits. A previous study showed that *E. pyrifoliae* did not grow as well as *E. amylovora* did when pear flowers were infected with *E. amylovora* and *E. pyrifoliae* and incubated at 23°C (Lehman et al., 2008). The real-time PCR revealed that the *E. amylovora* population grew to a larger size in the stigma than the *E. pyrifoliae* population and this growth has continued into the hypanthium. A previous study suggested that *E. amylovora* migrates through the stigma and inside the style to other plant tissues (van der Zwet and Keil, 1979). This is corroborated by a recent report, in which it has been displayed about the Type III secretion systems (T3SS), a bacterial secretion system that secretes their effector proteins into the host cell cytoplasm to facilitate the virulence mechanism (Pester et al., 2012). This model of infection is based on *E. amylovora* migrating down the flower stigma, swimming through nectar, and subsequently suppressing host defenses to invade the base of the flower through natural openings which is due to the stigma of the flower (Schachterle et al., 2022). Previous research has demonstrated that the moisture from rain or dew is required for *E. amylovora* cells to migrate from the stigma tip to the style of the flower, where the pathogen infects flowers through natural openings in the hypanthium, resulting in blossom blight (Thomson, 1986; Bayot and Ries, 1986). When shoot becomes infected, *E. amylovora* cells may migrate systemically through infected leaf and stem tissue toward the main trunk of the tree (Schachterle et al., 2022). As part of this migration, stem tissue can release oozing droplets in varying numbers and locations. Additional research has demonstrated that *E. amylovora* cells form biofilms in xylem vessels, obstructing water movement and causing wilting symptoms of fire blight (Peng et al., 2020). In contrast, Spinelli et al. (2007) found that *E. amylovora* migrated primarily into xylem vessels after reaching the nectar cup, despite the fact that the cortical parenchyma was also colonized in severely infected tissues.

In summary, we have identified two pathogens *E. amylovora* and *E. pyrifoliae* that cause fire blight and black shoot blight in apple orchards, in various cities across Korea, respectively. There is a trend in Korea toward more frequent and devastating outbreaks of fire blight than those of black shoot blight despite the erratic nature of fire blight, *E. amylovora* TS3128 was shown to be more motility than *E. pyrifoliae* at all temperatures tested, including 14°C, 18°C, and 27°C for swarming motility. The pathogenicity assay showed that both pathogenic bacteria caused severe symptoms, particularly on immature apple fruits at 27°C. We found that *E. amylovora* grew faster at lower temperatures, although it was more pathogenic than *E. pyrifoliae*. *E. amylovora* exhibited a greater movement and proliferation in apple flowers than *E. pyrifoliae*. Thus, in this study, we precisely evaluated the effect of temperature on the occurrence of these diseases in apple flowers, as well as the variations in black shoot blight. Understanding the characteristics of these two pathogens is necessary for developing science-based disease control policy in the field.

## Data Availability

The original contributions presented in the study are included in the article/Supplementary material, further inquiries can be directed to the corresponding author.
