# Supplementary material for: Evaluation of pathogenicity variation between two Erwinia species in apples and their population using a duplex real-time PCR method
Source: Front Microbiol. 2025 Feb 28;16:1514551. doi: 10.3389/fmicb.2025.1514551 (PMC11906481; doi:10.3389/fmicb.2025.1514551)
Supplement: Supplementary file 1 [file Image_1.pdf]

Evaluation of pathogenicity variation between two *Erwinia* species in apples and their population using a duplex real-time PCR method

Mi-Hyun Lee[1*], Kotnala Balaraju[1], Hyo-Won Choi[2], Yong Hwan Lee[1]

[1]Crop Protection Division, National Institute of Agricultural Sciences, Wanju, Republic of Korea
[2]Disaster Management Division, Rural Development Administration, Jeonju, Republic of Korea

[*]**Corresponding author:**
Dr. Mi-Hyun Lee
Tel.: +82-63-238-3282
Fax: +82-63-238-3838
E-mail: mihyun798@korea.kr
Running title: Pathogenicity variation of *Erwinia* species

Index scale	0	1	2	3	4	5
	0%	1-15%	16-30%	31-50%	51-75%	76-100%
Representative symptoms on leaves	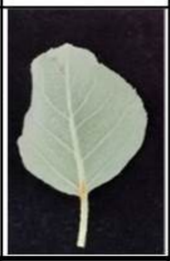	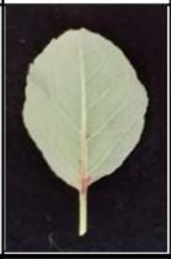	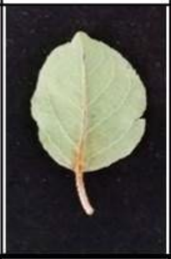	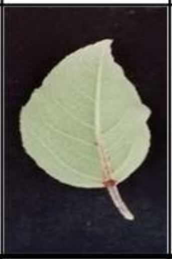	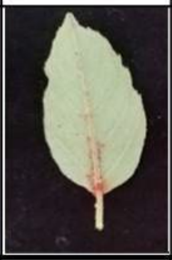	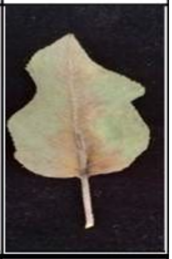
Index scale	0	1	2	3	4	5
Representative symptoms on flowers	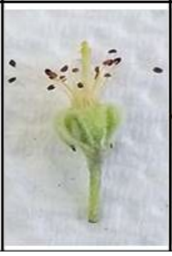	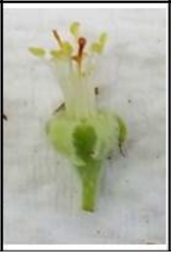	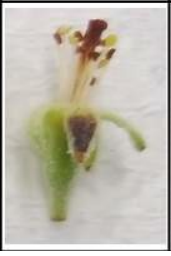	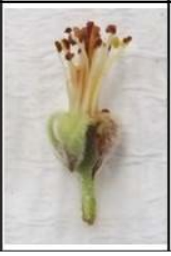	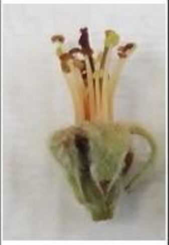	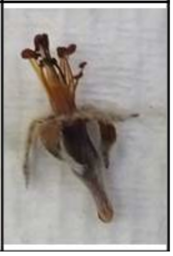

Supplementary Figure S1. Images representing the (A) disease index scale for symptoms on leaves ranging from 0 to 5 where 0= no symptoms, 1= lesions with <10% disease incidence, 2= lesions with 11 to 20%, 3= lesions with 21 to 40%, 4= lesions with 41 to 70%, 5= symptoms with >71 to 100%. (B) Disease index scale for symptoms on flowers where 0: no necrosis; 1: necrosis on the stigma; 2: necrosis visible on the stigma and hypanthium; 3: necrosis extending into the ovary, no farther than the widest point; 4: necrosis extending to the base of the ovary; 5: necrosis extending into the peduncle.
